# Patient experiences of colon capsule endoscopy: a qualitative study

**DOI:** 10.1177/26317745261433689

**Published:** 2026-03-25

**Authors:** Laura Jefferson, Holly Essex, Karl Atkin, Veronica Dale, Karen Bloor, Monica Haritakis, James Turvill

**Affiliations:** University of York, York, UK; University of York, York, UK; University of York, York, UK; University of York, York, UK; University of York, York, UK; York and Scarborough Teaching Hospitals NHS Foundation Trust, York, UK; York and Scarborough Teaching Hospitals NHS Foundation Trust, Wigginton Road, York, YO31 8HE, UK

**Keywords:** colon capsule endoscopy, colorectal cancer, colorectal polyps, diagnostic accuracy, gender differences, patient experience, qualitative research

## Abstract

**Background:**

Colon capsule endoscopy (CCE) was introduced by NHS England at scale during the COVID-19 pandemic to support colorectal diagnostics recovery. However, little was known about the patient experience of CCE.

**Objective:**

To explore the experiences of CCE during the NHS England pilot, to better understand what a patient-centered service for the future should look like.

**Design:**

A qualitative study to explore the patient and clinician experiences of CCE.

**Methods:**

Focus group discussions (*n* = 25 participants) and semi-structured interviews (*n* = 7), selected purposively on the basis of maximum variation, generated qualitative material exploring patient understanding, preferences and experiences. To place their experiences in context, we conducted asynchronous focus group discussions with clinicians (*n* = 16). Analysis was thematic.

**Results:**

Most patients valued CCE’s convenience and would repeat it, although for some their experiences would deter them from having the procedure again. Anxieties included fear when swallowing the capsule; concerns about it becoming stuck and/or whether the recording was working; what to do when the capsule was inside them; and worries about the novelty of the treatment. Women expressed greater discomfort, pain and anxieties during bowel preparation and the procedure than men. Participants highlighted the need for a more balanced description of the procedure than indicated in information leaflets and videos. They particularly felt the possibility of further investigations could be better explained. Healthcare professionals may underestimate patient concerns.

**Conclusion:**

CCE has the potential to expand colorectal diagnostic capacity but requires patients to be well informed and supported throughout the process. Gender differences in experiences of CCE are notable and could be partially addressed by providing more specific information, consistent with women’s experiences.

## Plain Language Summary

What is already known on this topic

- Colon capsule endoscopy (CCE) was introduced by NHS England as a pilot from 2020, but patient experiences remain underexplored. There are also no defined key performance indicators to inform the development of a high-quality, patient-centred CCE service for the NHS.

What this study adds

- This study finds patients view CCE positively and may prefer it over colonoscopy, though many report discomfort and anxiety. They also feel poorly informed.
- During the pandemic. healthcare professionals may have underestimated patients’ concerns about the procedure.

How this study might affect research, practice or policy

- Patients’ experience of CCE differs from colonoscopy.
- Relevant information, shared decision making and support are required throughout the CCE process.
- Information on the procedure may require rewriting to reflect the experiences of women.
- Acceptance of CCE, including at home or primary care use, depends on addressing these needs and managing expectations about potential follow-up procedures.

## Introduction

In June 2020, NHS England (NHSE) issued clinical guidelines supporting the use of colon capsule endoscopy (CCE) to enhance colorectal diagnostic capacity and support the restoration of endoscopy services during the COVID-19 pandemic.^1^ This guidance permitted CCE as an alternative to colonoscopy for patients referred with suspected colorectal cancer and later, those awaiting post-polypectomy surveillance. For those with suspected colorectal cancer, CCE was recommended if patients’ cancer risk was judged to be intermediate, based on a fecal immunochemical test (FIT) result of 10–100 µg Hb/g faeces.^2,3^ A clinical pilot to support rapid implementation of CCE was introduced through the Cancer Alliances, with structured training and clinical guidelines for patient selection, bowel preparation, CCE administration and post-procedure care. CCE had already been available in England as a colorectal diagnostic, prior to this pilot, however there was very little national experience of using it in these clinical settings or in wider standard care.

Colonoscopy, the current “gold standard” for colorectal cancer diagnosis, is an invasive procedure that can be uncomfortable and carries some risks.^4^ Discomfort, embarrassment and anxiety associated with colonoscopy may deter patients from seeking medical attention when they develop bowel symptoms.^5^ CCE, a camera in a pill swallowed by patients, uses a recording device attached to a belt to record optical images of the colon. It is diagnostically accurate, safe and assumed to offer a less invasive option.^6^ Emerging studies suggest less discomfort and embarrassment, potentially reducing the hesitancy some symptomatic patients may feel. CCE also appears well-tolerated by patients.^7,8^ It creates diagnostic capacity by sparing the need for onward colonoscopy for some, although completion rates are poor when compared to colonoscopy.^9,10^ A mixed methods study of patient perspectives in Scotland identified reduced travel and waiting times, along with the freedom to complete the procedure at home, as additional perceived benefits.^11^

In establishing a clinical service insights from patient perspectives are essential when refining CCE-based services. This includes determining whether CCE is regarded as an acceptable alternative to colonoscopy. Prior to wider adoption and to determine future care pathways, NHSE commissioned a pilot project exploring the patient experience of using CCE.^12^ This was designed to complement an investigation of patient safety and diagnostic accuracy.^8^ The qualitative study reported here sought to explore patients’ experiences of the CCE servicqes in greater depth, identify strategies to optimize potential benefits, mitigate CCE service risks and examine health professionals’ experiences of negotiating CCE use with patients. It complements the wider, structured, cross-sectional survey that was also conducted.^13^

## Methods

Consenting patients (aged > 17) who underwent CCE as part of the NHSE pilot (from April 2021 to March 2024) were eligible for inclusion in this qualitative study. Applying a pragmatic health services research approach to understand patient perceptions and experiences of this care pathway, we used focus group discussions to explore collective assumptions and enable participants to debate agreements/disagreements. In-depth interviews enabled us to locate our analysis in more personal experiences.

Patient participants included people referred with suspected colorectal cancer and those awaiting a three yearly post-polypectomy surveillance colonoscopy. There were no formal exclusion criteria for CCE, although NHS England issued guidance for purposive patient selection recommending that those unable to swallow the capsule, tolerate or comply with the procedure or who were at risk of stricture in the small or large bowel would be unsuitable for CCE. CCE was performed using the PillCamTM COLON 2 system (Medtronic.com) and was supported by patient facing product literature (medtronic.com/uk-en/pillcamcolon). All pilot sites followed the guidelines of the European Society of Gastroenterological Endoscopy (ESGE) for bowel preparation for CCE and received training provided by an approved online CCE reader training course (Imige Ltd).^14–16^ A few sites created their own patient-facing documentation. The median time for entry into the patient experience study had been 26 weeks after their investigation (range 2–92 weeks), this large window for recruitment reflecting the timeframe required to establish the study during the COVID-19 pandemic, once the NHS E pilot had begun. Patients, therefore, were reporting their retrospective reflections. Following the cross-sectional survey of patient experience, which followed guidance from the Consensus-Based Checklist for Reporting of Survey Studies (CROSS), this qualitative assessment of patient and professional experience used online focus group discussions and semi-structured interviews.^13,17^ The COREQ consolidated criteria for reporting qualitative research guidelines have applied (Supplemental Material).^18^

### Data collection

We conducted focus group discussions (*n* = 4) with patients to explore beliefs and assumptions about CCE before conducting interviews (*n* = 7) to provide a more in-depth and personal understanding of the process. We interviewed participants once. Initial focus groups were divided into (i) those who needed follow-up investigations and (ii) those who did not. Given the nature of CCE, we organized separate groups for men and women. Topic guides, informed by Health Literacy and Health Belief Models, explored (i) potential barriers and facilitators to the use of CCE and (ii) possible mediating influences, such as patient context and other characteristics (e.g., age and gender).^19,20^ We were particularly interested in how experiences evolved through the investigative pathway, from initial perceptions through to procedural and post-procedural experiences, and perceptions about the future use of CCE. Focus groups and interviews lasted 60–90 min. Only the participant/s and researchers were present.

With healthcare professionals, we conducted focus group discussions to explore experiences negotiating CCE use. To facilitate participation, we used asynchronous online discussions. These enable greater flexibility when capturing the views of busy health professionals.^21^ Over the course of 1 week (May 2023), targeted health professionals shared their experiences and perceptions via a closed online platform, with moderation by members of the research team, who also posed questions, based on health professionals’ responses.

The study gained NHS Ethics Approval (IRAS ID: 303921), obtained from East of England-Cambridge East Research Ethics Committee; REC reference: 21/EE/0234. All participants received an information leaflet and gave informed consent. The patient experience team within NHS England and the Public and Patient Voices (PPV) Forum offered a commentary on the research design and the interpretation of findings.

### Sampling and recruitment

We used purposive sampling from our structured, cross-sectional survey participants to capture a range of perspectives from men and women of varied ages; geographical locations; experiences of bowel disease; and the need for follow-up investigation (Table 1). Some had prior experience of colonoscopy, so were able to compare the two procedures. Healthcare professionals participated from pilot sites involved in the CCE patient experience cross-sectional survey.

**Table 1.** Participant characteristics.

Patient characteristics	
Gender	
Male	15 (47%)
Female	17 (53%)
Age (years)	
Median	66
IQR	9
Range	40–76
Ethnicity	
White British	31 (97%)
Black/African/Caribbean/Black British	1 (3%)
Time from CCE to data collection (months)	
Median	12
IQR	6
Range	2–20
Previous experience of colonoscopy	
Yes	18 (56%)
No	14 (44%)
Follow up investigations needed	
Yes	16 (50%)
No	16 (50%)
Health professional characteristics	Number
Role	
Consultant gastroenterologist	5
Clinical endoscopist	2
Clinical research nurse	4
Clinical nurse specialist	5
Location	
South of England	6
Midlands	2
North of England	8

### Analysis

Focus group discussions and the in-depth interviews were audio-recorded (with permission) and transcribed. Analysis, using NVivo 12 software, organized findings according to analytical and thematic headings.^22^ We applied an inductive approach, following stages of familiarization, to generate an initial coding framework before exploring relationships between codes. We collated codes into potential themes, which were discussed by the team and summarized using example quotations to demonstrate meaning. To clarify and explain recurring themes we compared different patient accounts. Analyses by age and sex explored further variations in experiences. Patient themes were then used to interrogate the accounts of healthcare professionals. The team conducting the interviews were a health economist with an interest in endoscopy (KB), a medical sociologist (KA) and experts in qualitative and quantitative health sciences research (HE and LJ). LJ and HE conducted analysis, supplemented by regular discussion with the wider team.

## Results

### Participant characteristics

Our 48 participants (see Table 1) included a convenience series of 32 patients (25 in focus group discussions and 7 in-depth interviews) who were recruited from 18 of the NHS Trusts in the CCE pilot. Fifteen men and 17 women made up the patient sample, the majority of whom were aged 60–69 (mean age 60, SD 9.19). Owing to the low number of non-white participants in our wider cross-sectional survey, only one patient from a minority ethnic group participated. Sixteen healthcare professionals (consultant gastroenterologists, clinical endoscopists and nurse specialists) participated in the asynchronous online discussion. Surgeons were invited but none recruited. Fieldwork occurred between January and May 2023 and was staggered to enable our patient experiences to inform the health professional discussions.

### Thematic findings

**Figure 1.**
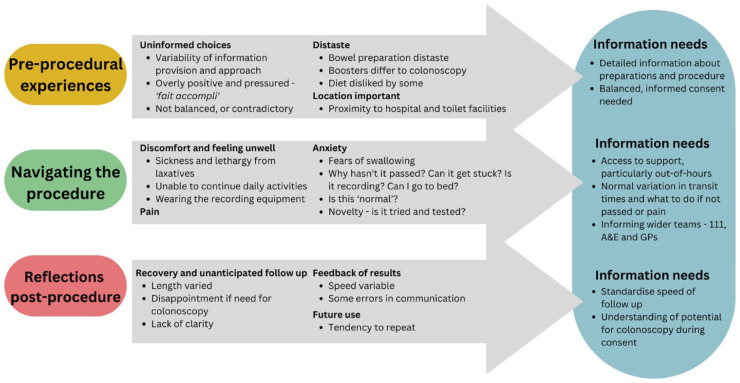
Overview of qualitative research finding.

Patient experiences were grouped under the thematic headings: “*pre-procedural experiences*,” “*navigating the procedure*” and “*reflections post-procedure*.” Recurring themes of discomfort, anxiety and the need for clear information occurred throughout these themes (see Figure 1). “*Future support needs*” are summarized in a final theme. Differences according to patient sex are described. No age differences were found.

#### Pre-procedural experiences

Patients had varied expectations prior to the CCE procedure, partly owing to previous experiences of colonoscopy and the information provided by clinical teams. Patients were generally optimistic about the prospect of CCE, especially those able to compare it with colonoscopy, who anticipated less discomfort. Others were reassured that the procedure could be done at home. Some (all male) associated CCE with the potential for less embarrassment.

##### Uninformed choices

Adequacy of patient information differed across sites. Some patients reported distrust because of what they regarded as the overly positive impression of CCE portrayed in information leaflets. Patients also described confusion, as they felt hospital and manufacturer leaflets provided contradictory information:

There were [discrepancies] between the information that was on the printed sheets that came from the hospital. . .and the manufacturer’s information that was in the [preparation medication] boxes. . .It was absolutely clear what to do and then when you read the other one, you thought, oh that’s not quite the same as on the other sheet. So, which one do I follow?

Male patient, 66, interviewee, no further investigation

Gender differences were apparent, with women more likely to report feeling poorly informed. Patients felt particularly ill-informed about the potential for further investigations. They also reported misunderstandings about the bowel preparation (described later). A few patients felt under pressure to accept CCE and those, who required further investigations, described regret:

I really wished I’d been offered a choice but I wasn’t. . .I ended up going for two colonoscopies [due to failure of preparations in the first] and I just thought well if I hadn’t have had the [CCE]. . .I actually ended up being really annoyed that I had to do it that many times. . .I didn’t get a choice.

Female patient, 40, further investigation needed

##### Bowel preparation (with which some included the procedural boosters)

All patients described an extreme aversion to the taste and quantity of bowel preparation taken prior to CCE. These observations, although generic to colonoscopy, seemed particularly unpleasant for CCE patients. This may be explained by the relative absence of discomfort in other aspects of CCE or the need for additional “boosters”:

My greatest problem was the medication. . .when you swallowed the pill you had to take [the booster] medication. Now they gave me anti-sickness tablets, but I was terribly, terribly, terribly sick. Really quite violently sick. And I was exhausted. And one thing that they said is to get it moving and you have to keep moving. Of course, you can’t go for a walk because you have to be near the loo. So, I was walking, bless him, with my husband up and down, up and down in my kitchen. . .the sickness had made me absolutely exhausted.

Female patient, 58, further investigation needed

The specialist diet needed before the preparations (as for colonoscopy) was also challenging, particularly for those with specific dietary needs. Gender differences were, however, evident, with men frequently minimizing distaste and more accepting of the medications used to prepare the bowel.

Distance from the hospital where the CCE was swallowed and rurality posed challenges due to the need for urgent access to toilets following bowel preparation. This would have been common to colonoscopy but nonetheless created anxieties:

I had to stop off at the doctor’s surgery. . .on the way, to use the toilet. . .I had spare clothes in the car in case there was an issue. . .it gets quite stressful because you don’t want to be late [for your appointment].

Female patient (rural area), 56, no further investigation

#### Navigating the procedure: unforeseen challenges

Patients reported initial anxiety about the capsule size but found it easier to swallow than anticipated. Some, however, described procedural discomfort, anxiety and pain, which they did not anticipate. Patients also reported discomfort from wearing the recording device, including when sleeping. Some identified a lack of guidance, which led to inappropriate clothing choices.

Patients described extreme lethargy and some felt unwell during the CCE procedure. They were not prepared for this. Many who had previous experience of investigations compared this negatively to colonoscopy:

I absolutely hated it. Then I was tired and I felt horrid the whole of the next day because of the amount of laxatives. . .In the end I went 40 hours. . .of not eating and that’s horrendous. You can eat like straightaway after colonoscopy.

Female patient, 40, further investigation needed

Some expressed anxiety about the CCE’s novelty, safety and diagnostic accuracy. A more common concern, however, was about whether the time taken to pass the capsule was normal. Women were more likely to worry about this and seek support from hospital teams, although it is unclear whether this is due to potential sex differences in transit times (delayed in six women and two men in our sample) or potential gendered norms around help-seeking. Information provided to patients did not address these anxieties.

While most patients (24/32) felt supported by clinical teams, men were more likely to report good support (15 men compared to 9 women). Inability to contact hospital teams out-of-hours heightened anxieties, particularly if the capsule had not passed:

I still was waiting for it to do that first move into the next part of your digestive system. So, I was panicking. The thing was [the nurse] was finishing at one, two o’clock. [The nurse] was like oh, you won’t need [to contact us] because that won’t happen, it’s only in very rare cases, and then it happens to you and you’re thinking I know she’s only going to be there till two.

Female patient, 50, no further investigation

Patients who passed the capsule quickly, however, were anxious about whether the capsule had recorded correctly or concerned that there may be an underlying medical reason causing more rapid passage than suggested on information leaflets.

##### Pain and out-of-hours support

Three patients (all women) experienced extreme pain during the CCE procedure and were unable to contact clinical teams for support, resulting in A&E attendance. Lack of awareness among A&E departments, GPs and the NHS helpline 111 during the NHSE pilot was a common problem.

I was nearly doubled over. . .my husband was really concerned. They’d given me a telephone number to contact. I’d tried our hospital where the procedure had gone ahead, nobody could be contacted. Not a soul. So, in the end phoned my GP. . .he said leave it with me, I’ll try and contact the hospital myself. In the meantime, I’ve still got this pain which was just. . .every few minutes it was just horrendous. . . [the GP] phoned back about five minutes later and he said I can’t contact anybody either. He said I suggest you go to A&E. [The A&E staff] hadn’t got a clue what I was talking about and kept looking at this pack around my. . .and the little computer that hangs off you. . .in the end I was in tears because of the pain. So, they had to give me. . .Oramorph. . .The pain was just horrendous.

Female patient, 74, further investigation needed

#### Reflections post-procedure

Reflecting on their experiences, some patients described the procedure as “*traumatic*,” believing it took a toll on their bodies, especially if a colonoscopy was needed shortly afterwards:

I’ve had malaria and this might have been a very close second to how horrendous I felt and then I couldn’t eat. . .I remember for the whole rest of that week I could only manage one meal a day. I couldn’t put much in my body without it coming straight through me

Female patient, 40, further investigation needed

Patients requiring follow-up colonoscopy voiced particular frustration and questioned the value of CCE. They felt inadequately informed about potential further investigations. Speed of follow-up varied by hospital, with some patients describing delays when accessing results, along with having insufficient information about rebooking procedures for colonoscopy. Health professionals raised similar issues, while contrasting this to the immediacy of colonoscopy results.

##### Future use

For patients and healthcare professionals, the perceived benefits of CCE, when compared to colonoscopy, included faster referral pathway, less time in hospital and not needing sedo-analgesia for the procedure. In our sample, 22/32 patients indicated they would choose to have a repeat CCE:

Nothing disappointed me at all. I was really quite chuffed with it. I thought it was a great system, I really did. I thought it was brilliant. It’s not just brilliant for the fact that it’s pretty non-invasive, it’s non-fussy.

Male patient, 60, no further investigation

Slightly more patients, who did not require follow-up investigations, said they would be comfortable repeating a CCE (13 vs 9). A similar proportion of male and female patients would also repeat the procedure (12 and 10). Nonetheless, gender differences occurred, when describing extreme negative experiences. This led to five women being strongly opposed to future CCE procedures. Other patients felt more confident in their knowledge of the procedure, having experienced it, and this would encourage their future use:

If you [understand potential challenges] then your outlook is completely different. Whereas if you think oh yeah, I’m going to be really lucky, having taken all [the laxatives] it’s going to go flying through me, I’m walking round all the time, it’s going to be really quick, you’re in cloud cuckoo land really. . .when you read [the information sheets] it made it sound like it was really unusual for you to get to the suppository. . .it’s not really unusual, is it.

Female patient, 56, no further investigation

#### Future support and information needs

Most participants were told they could continue with their “usual activities” during the preparations and procedure. They felt this was wrong. Some, for example, attempted to continue work and remarked on the difficulties this created. One patient soiled herself at the supermarket. Lack of information caused anxiety for patients, especially if they experienced negative effects:

I just felt so, it’s actually upsetting me at the moment recalling it. I’ve gotten over it, I will get over it. But I just feel that if anybody was having any other procedure, do you mean to say you get left on your own and you’ve got to deal with it? Or you go to A&E and they know nothing about it?

Female patient, 74, further investigation needed

Access to nursing team support had a positive impact on patients’ experiences. Health professionals acknowledged that having limited time to discuss CCE with patients, particularly if they are part of a suspected colorectal cancer referral pathway was a challenge facing nursing teams:

Sometimes it takes too long to answer all their questions in order to help them make a choice between CCE and colonoscopy which creates a challenge time wise, particularly in a busy two week wait [suspected colorectal cancer referral] clinic.

Consultant gastroenterologist

Health professionals described strategies to overcome patient anxieties, including booking longer appointments, although in locations with high patient volumes, professional capacity to provide such support was limited. One site, however, had developed their own information video and leaflet to improve communication. Table 2 provides integrated recommendations from both patient and health professionals insights.

**Table 2.** Strategies to support the patient experience.

**1. Planning** • Careful patient selection, with pre-assessment of any contraindications or communication barriers **2. Initial consultation** • Sufficient time to enable full explanation of the procedure beforehand and time to ask questions • Bringing a friend or family for support • Offering a window of time for arriving at appointments to reduce the pressure of travel time after taking preparations **3. Information sharing methods** • Novel approaches to information sharing, including videos developed in a balanced manner • Information about preparations and the period after swallowing the CCE • Open sharing of potential challenges and when to ask for help • Insights into transit times (and breadth of experiences, potentially varying by sex) • How to sleep with the device **4. Support and reassurance** • Sharing contact details for support needs, particularly out-of-hours • Proactively telephoning patients during procedure, particularly when boosters may be required to ensure understanding • Explaining that CCE is not a novel procedure • Reassurance that if further procedure is needed, patients would be booked onto appropriate list to enable polyp removal. **5. Informing wider health professionals** • Information sharing with A&E and GP teams

## Discussion

Most patients reported a positive experience of CCE. Some, however, described the experience as painful and distressing. Some also expressed frustration about the need for follow-up colonoscopy. This is an especially important consideration as approximately half of CCE respondents in our survey needed further investigations.^13^ Further, many patients felt inadequately informed, when choosing whether CCE was the best investigation for them and in preparing them for the procedure. This contrasts with health professionals’ belief that patients made informed choices. The context of the COVID-19 pandemic may have had some bearing on this. However, many patients found information leaflets confusing and lacked information about what to expect. This caused anxiety, especially when patients had negative experiences or worried about the capsule transit time.

Our findings report some similarities with patient experiences of colonoscopy. Communication is identified as especially important, particularly when managing expectations of the procedure.^23^ However, our sample raised some unfavorable comparisons with colonoscopy. CCE patients were more likely to say that the information received or the discussions that occurred offered insufficient preparation for the procedure. Troublesome bowel preparation, while common to CCE and colonoscopy, seemed more challenging for CCE patients.^24,25^ Discomfort, although a feature common to CCE and colonoscopy, assumed a particular meaning in patients having a CCE and reflected a broader range of anxieties, experienced over a longer period of time.^24^ It is possible that bowel preparation, combined with the need to take boosters, came to represent the major challenge in patients undergoing CCE, since the procedure itself caused little pain. However, there appears to have been greater potential for uncertainty in patients undergoing CCE, which was made worse by the wait for results. Women, having CCE expressed greater anxieties than men, had particular difficulties relating their experience to the information they received. Men were more likely to believe that CCE was less embarrassing, when compared to women. Gender differences are, of course, a feature of patient-reported experiences of colonoscopy. A systematic review of colonoscopy found that pain and discomfort were frequently reported during and after the procedure, and that, women reported a higher degree of abdominal pain, more complications and greater difficulty sleeping/longer day disturbance in the days before and after the procedure.^25^ Perhaps gender specific guidance should be developed for those having CCE. Nonetheless, patients appreciated the convenience of CCE and generally found it a tolerable intervention, which many–despite the challenges–would repeat.

### Implications for practice, policy and research

This study, conducted during the introduction and rapid expansion of CCE use during the COVID-19 pandemic, offers valuable insights into practical improvements required to support the development of a future colorectal diagnostic pathway. There was a possible disconnect between the perceptions of healthcare professionals and those of patients, with healthcare professionals less likely to be aware of patient concerns. This must be addressed. Examples of good practice were, however, described by patients and professionals. This involved supporting informed consent and providing ongoing support to patients through the CCE journey. Support, however, varied, which professionals believed was affected by local demand. Discussion about the potential need for further procedures after CCE, such as a colonoscopy, which may have been constrained during the COVID-19 pandemic, should be an important part of patient discussions, as should peri-procedural and out-of-hours support.

These findings, alongside our wider, structured, cross-sectional survey, highlight significant differences in the experience of CCE between men and women.^13^ This would suggest a need for a more patient-centered approach when negotiating the use of CCE and highlights a need for consistent quality standards, such as Patient Reported Experience Measures (PREMs) for CCE. Discomfort, pain and anxiety were greater for women during bowel preparation and during the procedure. Explaining the likelihood of these experiences would better prepare female patients, support decision making and manage expectations. Beyond this it is also important for service providers (and research) to explore the reasons for these different experiences, particularly since they could impact upon how a future CCE service is implemented.

Our sample included patients from geographically dispersed locations with varying levels of infrastructure support for the delivery of CCE; and differing patient groups in terms of underlying pathology and need for further procedures.

### Limitations

There were some patient groups that we were unable to engage, including patients who had turned down the procedure. Further, while we had intended to include an ethnically diverse sample, we were unable to achieve this given the low numbers of ethnic minority participants taking part in the survey. It is difficult to understand the reasons for this and we are unable to assess whether they were less likely to respond to the survey, less likely to choose CCE or less likely to be offered CCE. While participation in research is lower among ethnic minority groups, for varied reasons, it is also possible they are less likely to be offered novel interventions.^26^ This requires further investigation, to avoid potential inequities, not least because the near to home options for CCE delivery may be attractive to some.

Our research took place during a time of rapid development in the setting of the COVID-19 pandemic and a formal power analysis for a sample size calculation was not appropriate. Nevertheless, we believe that the experiences described are relevant and should inform future CCE provision.

We found it difficult to engage consultant surgeons and gastroenterologists and as a result the majority of the perspectives have been those of the participating patients. Instead, health professional participation tended to come from the nursing teams, although they may have more patient contact, perhaps greater awareness of the patient perspective and more experience of capsule service delivery.

There are several possible explanations for the differing views of patient and healthcare professionals. There may have been a bias toward greater participation in the online focus group discussion from health professionals at more established sites with greater engagement in CCE support. Likewise, there may have been a bias toward patients with more negative viewpoints wishing to share their experiences. Conversely, notions of being a “good patient” may result in patients being unlikely complain to clinical team, although some patients said no one asked them for feedback on the procedure.

## Conclusion

Most patients reported a positive experience of CCE, though some found it challenging. Many highlighted gaps in the information provided and the support available during and after the procedure. To strengthen CCE as an alternative colorectal diagnostic, patient information and support must be improved, potentially incorporating PREMS into service evaluation to identify these gaps and develop an enhanced patient experience of this innovative diagnostic technology.^27^

## Supplemental Material

sj-docx-1-cmg-10.1177_26317745261433689 – Supplemental material for Patient experiences of colon capsule endoscopy: a qualitative study Supplemental material, sj-docx-1-cmg-10.1177_26317745261433689 for Patient experiences of colon capsule endoscopy: a qualitative study by Laura Jefferson, Holly Essex, Karl Atkin, Veronica Dale, Karen Bloor, Monica Haritakis and James Turvill in Therapeutic Advances in Gastrointestinal Endoscopy

## Declarations

**Ethics approval and consent to participate:** The study gained NHS Ethics Approval from East of England—Cambridge East Research Ethics Committee (IRAS ID: 303921) and was sponsored by York and Scarborough Teaching Hospitals NHS Foundation Trust. Oversight was provided by an Expert Advisory Group. All participants provided written consent prior to taking part in this study.

**Consent for publication:** All participants provided written consent for the publication of this study, including pseudo anonymised patient quotations.

**Patient and public involvement:** PPI support was provided by the patient experience team within NHS England and the Public and Patient Voices (PPV) Forum.

## References

[1] NHS England. Clinical guide for triaging patients with lower gastrointestinal symptoms. NHS England, https://www.nice.org.uk/media/default/about/covid-19/specialty-guides/triaging-patients-with-lower-gi-symptoms.pdf, 2020.

[2] Loveday C Sud A Jones ME , et al. Prioritisation by FIT to mitigate the impact of delays in the 2-week wait colorectal cancer referral pathway during the COVID-19 pandemic: a UK modelling study. Gut 2021; 70(6): 1053–1060. 32855306 10.1136/gutjnl-2020-321650 PMC7447105

[3] National Institute for Health and Care Excellence. Suspected cancer: recognition and referral. National Institute for Health and Care Excellence, https://www.nice.org.uk/guidance/ng12, 2021.

[4] McLachlan S-A Clements A Austoker J. Patients’ experiences and reported barriers to colonoscopy in the screening context—a systematic review of the literature. Patient Education Counseling 2012; 86(2): 137–146. 21640543 10.1016/j.pec.2011.04.010

[5] Yang C Sriranjan V Abou-Setta AM , et al. Anxiety associated with colonoscopy and flexible sigmoidoscopy: a systematic review. Am J Gastroenterol 2018; 113(12): 1810–1818. 30385831 10.1038/s41395-018-0398-8 PMC6768596

[6] Thygesen MK Baatrup G Petersen C , et al. Screening individuals’ experiences of colonoscopy and colon capsule endoscopy; a mixed methods study. Acta Oncologica 2019; 58(Suppp 1): S71–S76. 10.1080/0284186X.2019.1581372 30821625

[7] Ismail MS Murphy G Semenov S , et al. Comparing colon capsule endoscopy to colonoscopy; a symptomatic patient’s perspective. BMC Gastroenterol 2022; 22(1): 31. 35073873 10.1186/s12876-021-02081-0 PMC8785487

[8] Turvill J Haritakis M Pygall S , et al. Multicentre study of 10,369 symptomatic patients comparing the diagnostic accuracy of colon capsule endoscopy, colonoscopy and CT colonography. Alimentary Pharmacol Therap 2025; 61(9): 1532–1544. 10.1111/apt.70046 PMC11981550 40012235

[9] Ravindran S Bassett P Shaw T , et al. National census of UK endoscopy services in 2019. Frontline Gastroenterol 2021; 12(6): 451–460. 34712462 10.1136/flgastro-2020-101538 PMC8515281

[10] MacLeod C Hudson J Brogan M , et al. ScotCap–a large observational cohort study. Colorectal Dis 2022; 24(4): 411–421. 34935278 10.1111/codi.16029 PMC9305214

[11] Bond S Kyfonidis C Jamieson M , et al. Evaluation of an innovative colon capsule endoscopy service in Scotland from the perspective of patients: mixed methods study. J Med Internet Res 2023; 25: e45181. 10.2196/45181 PMC10148218 37058337

[12] NHS England. Harnessing innovation in cancer care: Pillcam Colon Capsule Endosopy, https://www.england.nhs.uk/cancer/harnessing-innovation-in-cancer-care/, 2021.

[13] Dale V Essex H Bloor K , et al. Patient experience of colon capsule endoscopy in clinical practice: a structured, comparative patient survey. BMJ Open Gastroenterol 2025; 12: e001944. 10.1136/bmjgast-2025-001944 PMC12519712 41087040

[14] JAG Endoscopy Training System: Colon Capsule Endoscopy Training Position Statement. https://www.thejag.org.uk/Downloads/JAG/JAG%20certification/Colon%20Capsule%20certification%20-%20JAG%20position%20statement%20-%20%20Jan%202022.pdf, 2022.

[15] What is the Institute for Minimally Invasive Gastroenterology (IMIGe)? https://imige.co.uk/

[16] Spada C Hassan C Galmiche JP , et al. Colon capsule endoscopy: ESGE guideline. Endoscopy 2012; 44: 527–536. 22389230 10.1055/s-0031-1291717

[17] Sharma A Minh Duc NT Luu Lam Thang T , et al. A Consensus-Based Checklist for Reporting of Survey Studies (CROSS). J Gen Intern Med 2021; 36(10): 3179–3187. 33886027 10.1007/s11606-021-06737-1 PMC8481359

[18] Tong A Sainsbury P Craig J. Consolidated criteria for reporting qualitative research (COREQ): a 32-item checklist for interviews and focus groups. Int J Quality Health Care 2007; 9(6): 349–357. 10.1093/intqhc/mzm042 17872937

[19] Baker DW. The meaning and the measure of health literacy. J General Int Med 2006; 21(8): 878–883. 10.1111/j.1525-1497.2006.00540.x PMC1831571 16881951

[20] Champion VL Skinner CS. The health belief model. In: Glanz K Rimer BK Viswanath K , editors. Health Behavior and Health Education: Theory, Research, and Practice, 2008, vol. 4, pp. 45–65.

[21] Gordon AR Calzo JP Eiduson R , et al. Asynchronous online focus groups for health research: case study and lessons learned. Int J Qualitative Methods 2021; 20. 10.1177/1609406921990489 PMC8856649 35185443

[22] Braun V Clarke V. Conceptual and design thinking for thematic analysis. Qualitative Psychol 2022; 9(1): 3–26.

[23] Tierney M Bevan R Rees CJ , et al. What do patients want from their endoscopy experience? The importance of measuring and understanding patient attitudes to their care. Frontline Gastroenterol 2016; 7: 191–198. 27429733 10.1136/flgastro-2015-100574 PMC4941156

[24] Decruz GM Ng CH Lim KT , et al. Afterthoughts on colonoscopy. Was it that bad? J Med Screening 2020; 28(2): 63–69. 10.1177/0969141320923381 32438893

[25] Kayal G Kerrison R Hirst Y , et al. Patients’ experience of using colonoscopy as a diagnostic test after a positive FOBT/FIT: a systematic review of the quantitative literature. BMJ Open 2023; 13: e071391. 10.1136/bmjopen-2022-071391 PMC10514644 37734900

[26] Pardhan S Sehmbi T Wijewickrama R , et al. Barriers and facilitators for engaging underrepresented ethnic minority populations in healthcare research: an umbrella review. Int J Equity Health 2025; 24(1): 70. 40075407 10.1186/s12939-025-02431-4 PMC11905581

[27] Dobson C Haworth E Sharp L , et al. “You don’t know what to expect”: unmet needs in patient experience of capsule endoscopy. Frontline Gastroenterol 2025; 0: 1–8.

